# Behavioral abnormalities in C57BL/6 mice with chronic ulcerative colitis induced by DSS

**DOI:** 10.1186/s12876-023-02718-2

**Published:** 2023-03-23

**Authors:** Yuxin Zhou, Gang Ji, Xiaoyi Yang, Zhenhua Chen, Liangliang Zhou

**Affiliations:** School of Pharmacy, Jiangxi Science & Technology Normal University, Nanchang, 330013 PR China

**Keywords:** DSS, Ulcerative colitis, Behavioral abnormalities

## Abstract

**Background:**

Clinical epidemiological studies have found that some patients with ulcerative colitis (UC) are prone to mental disorders. DSS-induced acute and chronic UC models are often used to evaluate the efficacy of anti-UC drugs. However, whether DSS has an effect on mouse behavior has not been reported.

**Methods:**

Acute and chronic UC models were induced by 3% DSS and 1.5% DSS, respectively. The bloody stool, the changes in the colon length, and histopathological changes in the colon were used to evaluate the success of the animal model. The behavior of mice was evaluated by open field experiment, tail suspension experiment and Sucrose preference test.

**Results:**

The weight of mice in 3% DSS group decreased significantly, the DAI score increased significantly, the colon length of mice was significantly shortened, and the structure of colonic crypts was abnormal, which showed inflammatory cell infiltration and shrinkage of crypts. Compared with the control group, the immobility time of 3%DSS group mice in the tail suspension test and forced swimming test had no effect, the number of running and grooming times was significantly reduced, and there was no significant difference in the number of standing times. No abnormality was observed in HE staining of the hippocampus. However, in 1.5% DSS-induced chronic UC model, behavioral and hippocampal abnormalities were observed not only UC symptoms.

**Conclusions:**

The acute UC model induced by 3% DSS has certain influence on the behavior of mice, but the mental state of mice is normal, which may be the abnormal behavior caused by UC symptoms; However, the chronic UC model induced by 1.5% DSS has a significant effect on the behavior of mice, and the mice have mental disorders, which are caused by mental disorders.

## Introduction

Ulcerative colitis (UC) is a chronic non-specific intestinal inflammatory disease characterized by diarrhea, purulent bloody stool and abdominal pain [1]. The rectum and colon are the main sites of onset. The course of UC is long, the recurrence rate is high, and it is easy to develop into colorectal cancer [2, 3]. Worldwide, the incidence of UC is still on the rise. The incidence rate in North America and Europe is the highest in the world, but it has stabilized [4]. However, the incidence rate of UC is increasing in developing countries. In China, people’s living standard is rising, and the incidence rate of UC has increased dramatically, which is more and more harmful to people’s health and living standard [5].

The pathogenesis of UC is complex and unclear. Many factors such as geography, age, gender, genetics, immunity and mental stress are involved in the pathogenesis of UC [6, 7]. Moreover, these pathogenic factors interact with each other, resulting in a low clinical cure rate and easy recurrence of UC. More and more clinical studies have found that the incidence of mental disorders in patients with UC is much higher than that in patients with chronic intestinal diseases without inflammatory bowel diseases [8, 9]. The incidence of depression (16.7%) and anxiety disorder (35.1%) in patients with UC are 2–4 times that of depression (5.9%) and 2–5 times that of an anxiety disorder (7.3%) [10]. At the same time, compared with the remission stage of UC, the incidence of anxiety and depression in patients with the active stage increased significantly [11]. In addition, a poor mental state can also increase the risk of recurrence of UC. Clinicians found that the use of antipsychotic drugs paroxetine, sertraline and Deanxit combined with mesalazine in the treatment of UC is better than mesalazine alone, especially for UC patients with mental abnormalities [12]. At the same time, appropriate psychological intervention for UC patients can effectively alleviate the concurrent mental problems, which are conducive to the recovery of UC. These results suggest that the pathogenesis of UC is associated with mental factors.

Since Kirsner successfully induced colitis model in rabbits for the first time in 1957, for more than 60 years, researchers have tried to establish different types of animal models with mice, rats, rabbits, monkeys and sheep as research objects to study the pathological mechanism and pharmacodynamic screening of UC [13–17]. C57BL / 6 mice are the main model animals because of their small size, convenient feeding and histopathology more similar to human UC [18]. Genetic engineering, drug induction, adoptive transfer, bacteria and spontaneous are the most common methods to establish the C57BL / 6 mouse UC model [13, 17]. The UC model induced by dextran sulfate sodium salt (DSS) is most commonly used to evaluate the efficacy of anti-UC drugs [19]. Because of its simple operation, short modeling time, long lesion duration, obvious acute inflammation period, and low concentration repeated stimulation to induce chronic inflammation. However, whether DSS induced UC model can be applied to study the mechanism of antipsychotic drugs in the treatment of UC has not been reported. 3% DSS and 1.5% DSS were used to induce the acute UC model and chronic UC model respectively, and their effects on the behavior of C57BL / 6 mice were investigated.

## Materials and methods

### Chemicals and reagents

Dextran Sodium Sulfate (DSS) was purchased from Regent Technology (HK) Co., Ltd. (serial number: 11032–220); Fecal occult blood qualitative test kit was purchased from Shanghai yuanye Bio-Technology Co., Ltd (serial number: D08GR170742); purified water was purchased from Hangzhou Wahaha Group Co., Ltd. (serial number: 202110227215NC).

### Animals

The experiment was reported according to the ARRIVE guidelines 2.0. The protocol was approved by the Animal Experimentation Ethics Committee of Jiangxi Science and Technology Normal University (Approval ID: JXSTNU-2021–0019). After referring to the AVMA animal euthanasia guidelines (2020), mice were killed by cervical dislocation. Healthy adult male C57BL/6 mice (6–8 weeks) were issued by Changzhou CAWENS Laboratory animal co., Ltd. (Approval ID: SCXK (su) 2016–0010). The animals were maintained the following conditions: room temperature (22 ± 2℃); humidity (50 ± 5%); and a 12 h light/dark cycle. During this period, food and water were provided ad libitum.

### Animal experimental designs

#### Induction of acute UC model

**Fig. 1:**
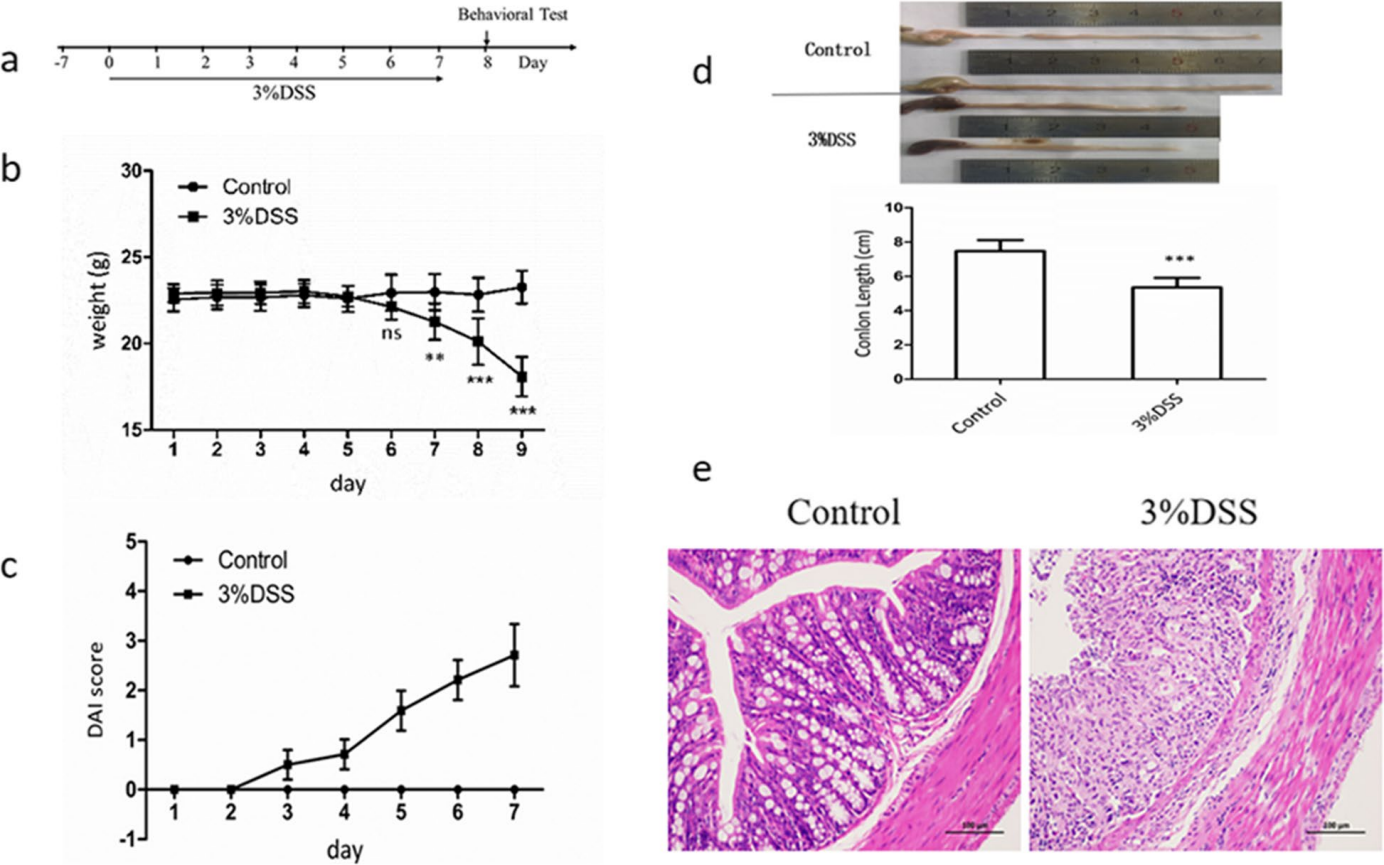
DSS-induced acute ulcerative colitis. C57BL/6 mice were fed 3% (DSS) for 7 days and then tested for behavior. Mice were sacrificed at the end of the experiment, and brains and colons were collected. **a** Experimental procedure, **b** body weight change, **c** disease activity index (DAI), **d** colon length, **e** colon HE staining(200 ×). Data are expressed as mean ± SD of ten mice in each group. *P < 0.05, **P < 0.01 and *** < 0.001vs. Control group

Twenty C57BL/6 mice were randomly divided into 2 groups, namely Control group and 3%DSS group. 3% DSS groups were given 3% g/mL DSS solution to drink water freely for 7 days. Fresh DSS solution was replaced, every other day, and the Control group drank sterile water freely. The body weight, stool characteristics and occult blood of mice were observed every day, and the disease activity index (DAI) was calculated according to (Table 1). The experimental flow chart is shown in (Fig. 1a).

**Table 1:** Disease Activity Index (DAI) Scores. DAI score = (weight loss rate score + stool trait score + occult blood degree score)/3

weight loss%	Stool traits	fecal occult blood / gross blood in the stool	scores
0	normal	normal	0
1–5	loose	occult blood weakly positive	1
5–10	soft stool	occult blood strongly positive	2
10–15	loose stool	gross blood in the stool	3
> 15	diarrhea	massive bleeding in the stool	4

#### Induction of chronic UC model

**Fig. 2:**
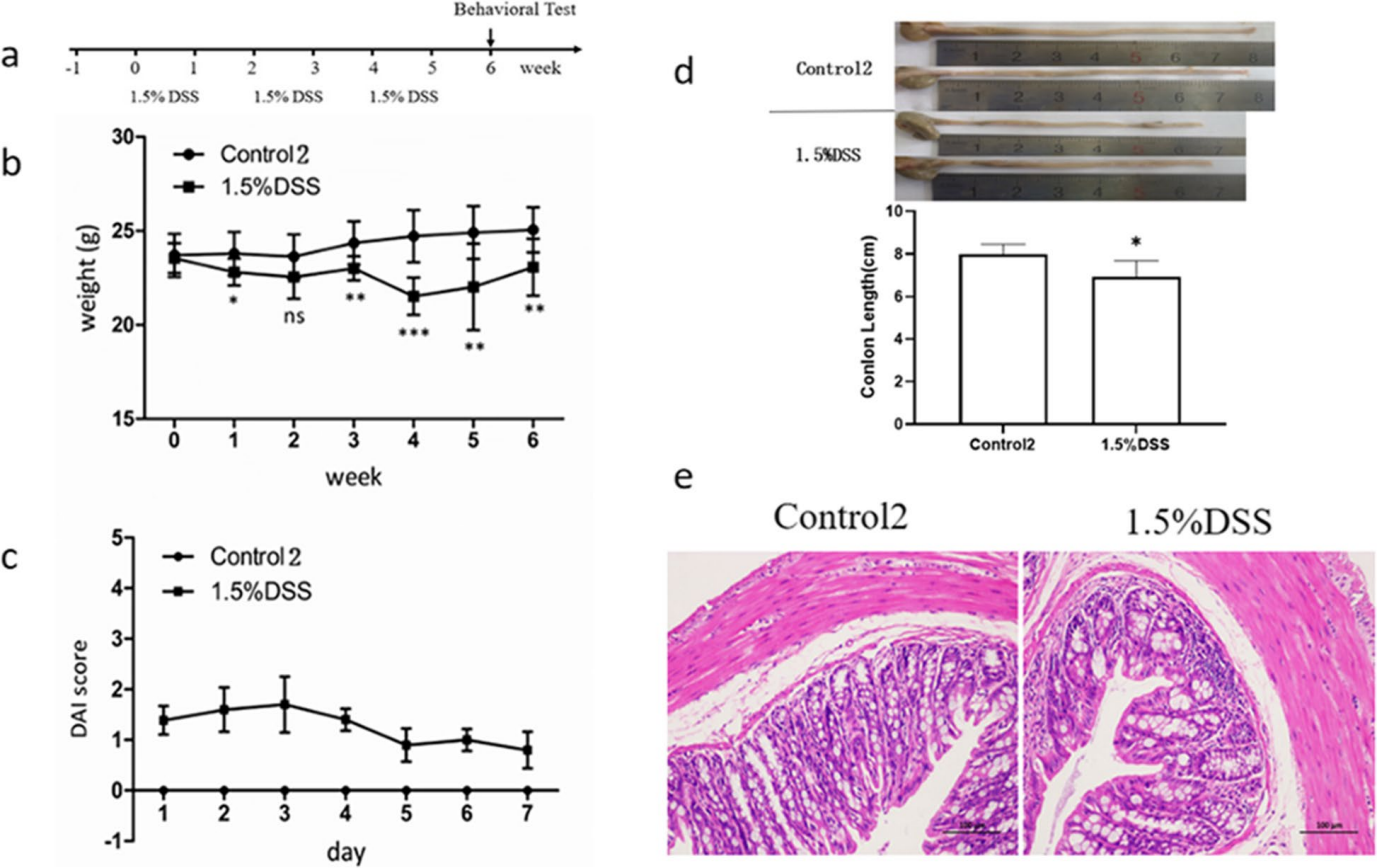
DSS-induced chronic ulcerative colitis. C57BL/6 mice were fed 1.5% (DSS) for 7 days followed by purified water for 7 days, repeated 3 times, and then tested for behavior. Mice were sacrificed at the end of the experiment, and brains and colons were collected. **a** Experimental procedure, **b** body weight change, **c** disease activity index (DAI), **d** colon length, **e** colon HE staining(200 ×). Data are expressed as mean ± SD of ten mice in each group. *P < 0.05, **P < 0.01 and *** < 0.001vs. Control group.Data are expressed as mean ± SD of ten mice in each group. *P < 0.05 and**P < 0.01 vs. Control2 group

Twenty C57BL/6 mice were randomly divided into 2 groups, namely Control2 group and 1.5%DSS group. 1.5% DSS solution was continuously drunk for 7 days. Fresh DSS solution was replaced every other day, and pure water without additive was given for 7 days, and then the above DSS/purified water was drunk for 3 consecutive cycles. The body weight, stool characteristics and occult blood of mice were observed every day, and the disease activity index (DAI) was calculated according to (Table 1). The experimental flow chart is shown in (Fig. 2a).

### Hematoxylin–eosin staining

The colon and brain were fixed with 4% formalin, stained with hematoxylineosin (H&E), and then reviewed using a FV1000 laser confocal inverted microscope.

### Behavioral evaluations

Open field experiment: The experimental device was improved according to the method established by Archer [20]. Mice were placed in the center of an observation box (80 cm × 80 cm × 40 cm) for adaptive activity 2 min and then recorded the number of trails crossed by running from the bottom center, the times of standing on the hind legs (including standing on the upright wall and standing freely) and the times of hairdressing were recorded in the darkroom and quiet environment.

Tail suspension experiment: The white adhesive tape was used to attach to the tail suspension bracket about 2 cm from the tail tip of the mice. The head of the mouse was suspended downward in the box, about 6 cm from the bottom of the box. In the soundproof black box, the activity of mice was recognized by a signal acquisition system. After adjusting for 2 min, the immobility time of mice in 4 min was recorded.

Sucrose preference test: Before starting the experiment, each group of mice was given two bottles of 1% sucrose aqueous solution, and after 12 h of free drinking water, one of the bottles was replaced with purified water, and then free water for 12 h. After the mice adapted, each group of mice was placed with sucrose water and purified water, marked, and the positions of the two bottles of water were changed every 8 h, and the remaining volume was recorded 24 h later [21].

### Statistical analysis

All data were shown as mean ± SD, and n represents the number of animals. Statistical analysis of the results was performed using the unpaired T test (two-tailed). Data were analyzed by GraphPad Prism software (version 9.0; GraphPad Software, Inc., La Jolla, CA). ns represent there is no significant difference. *P* < 0.05, 0.01, and 0.001 were considered statistically significant.

## Results

### 3%DSS induced acute UC model

In order to study whether there are behavioral abnormalities in DSS induced acute UC mice, 3% DSS solution was used to prepare acute UC model. Compared with the normal control group, after drinking the aqueous solution containing 3% DSS for 7 consecutive days, the weight of mice decreased significantly (Fig. 1b), the DAI score increased significantly (Fig. 1c), and the colon shortened significantly (Fig. 1d).

The results of HE staining showed that the mucosa of mice in 3%DSS group was seriously damaged, with a large number of inflammatory cell infiltration and crypt loss (Fig. 1e). This indicated that 3% DSS free drinking water for 7 days can well induce acute UC symptoms in C57BL / 6 mice.

### 1.5% DSS induced chronic UC model

Most patients with ulcerative colitis are chronic inflammatory bowel disease [22]. Low dose DSS is often used to induce chronic colitis model. The chronic UC model induced by 1.5% DSS / purified water for 3 cycles was used to investigate whether there were behavioral abnormalities in DSS induced chronic UC model mice. Compared with the control group, the body weight decreased (Fig. 2b), the DAI score increased, and the symptoms were mild despite diarrhea and bloody stool in 1.5% DSS group mice (Fig. 2c). However, the colon of mice was still significantly shortened (Fig. 2d), with inflammatory cell infiltration and recess loss (Fig. 2e). This indicated that 1.5% DSS can induce chronic UC symptoms in C57BL / 6 mice.

### Effect of 3% DSS on behavior in mice

**Fig. 3:**
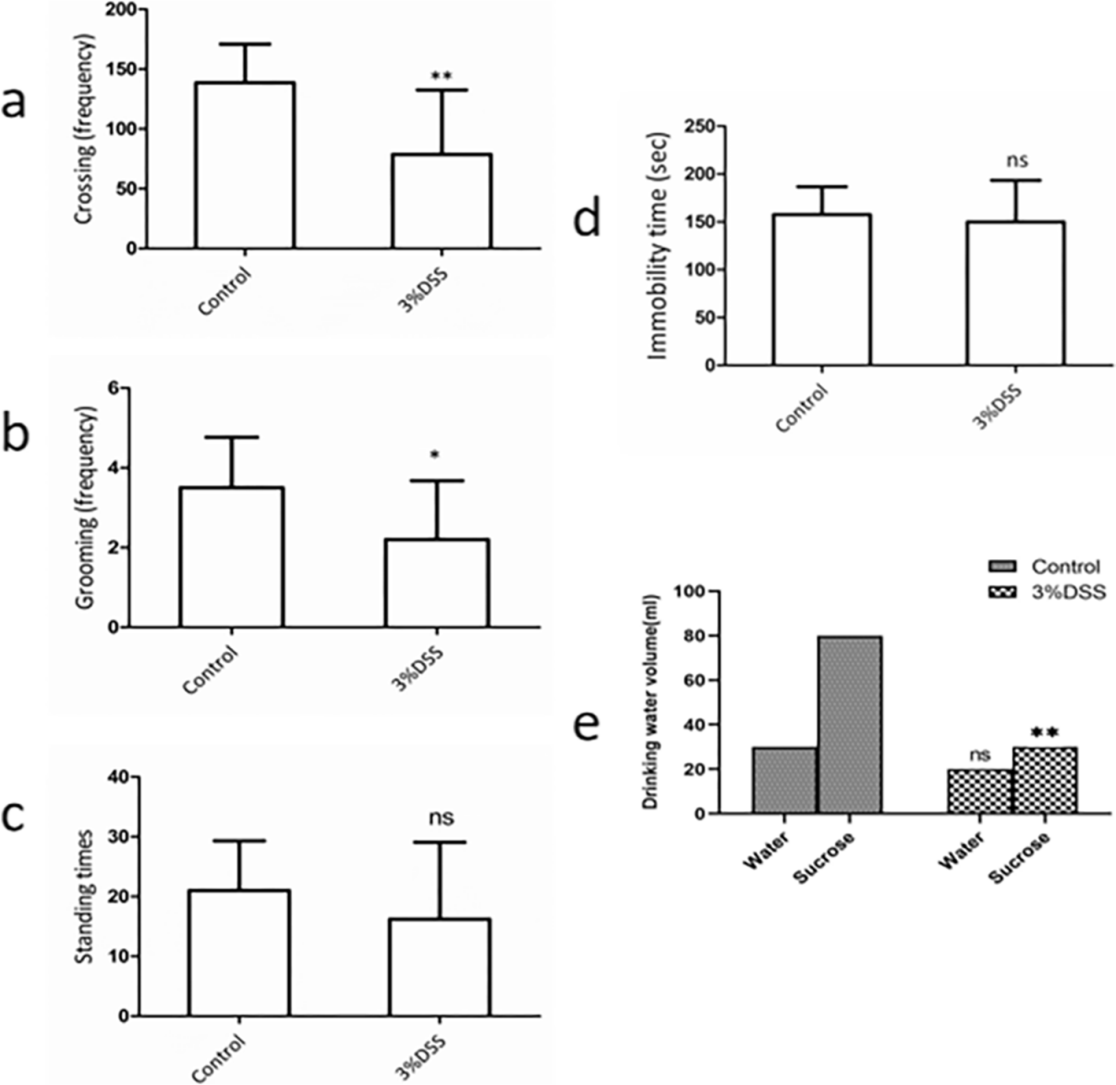
Behavior of mice in acute UC model group. After the UC model was established, C57BL/6 mice were detected open field experiment, tail suspension experiment and sucrose preference experiment. **a** Running times, **b** grooming times, **c** standing times, **d** tail suspension resting time,  **e** sucrose preference. Data are expressed as mean ± SD of ten mice in each group. *P < 0.05 and **P < 0.01vs. Control group

Open field experiment, Tail suspension experiment and Sucrose preference are proved to be effective and reliable method to evaluate behavioral abnormalities in animal models of depression [23–25]. In the open field experiment, the number of runs and grooming decreased, rather than the number of standing times, in the DSS group, the significant difference of immobility time was also not found in the tail suspension experiment(Fig. 3).In the sucrose preference experiment, the consumption of purified 8 water and sucrose water was significantly reduced in the 3% DSS group. These results indicated that the acute UC model induced by 3% DSS has a certain effect on the behavior of mice.

### Behavioral changes in chronic UC model induced by 1.5% DSS

**Fig. 4:**
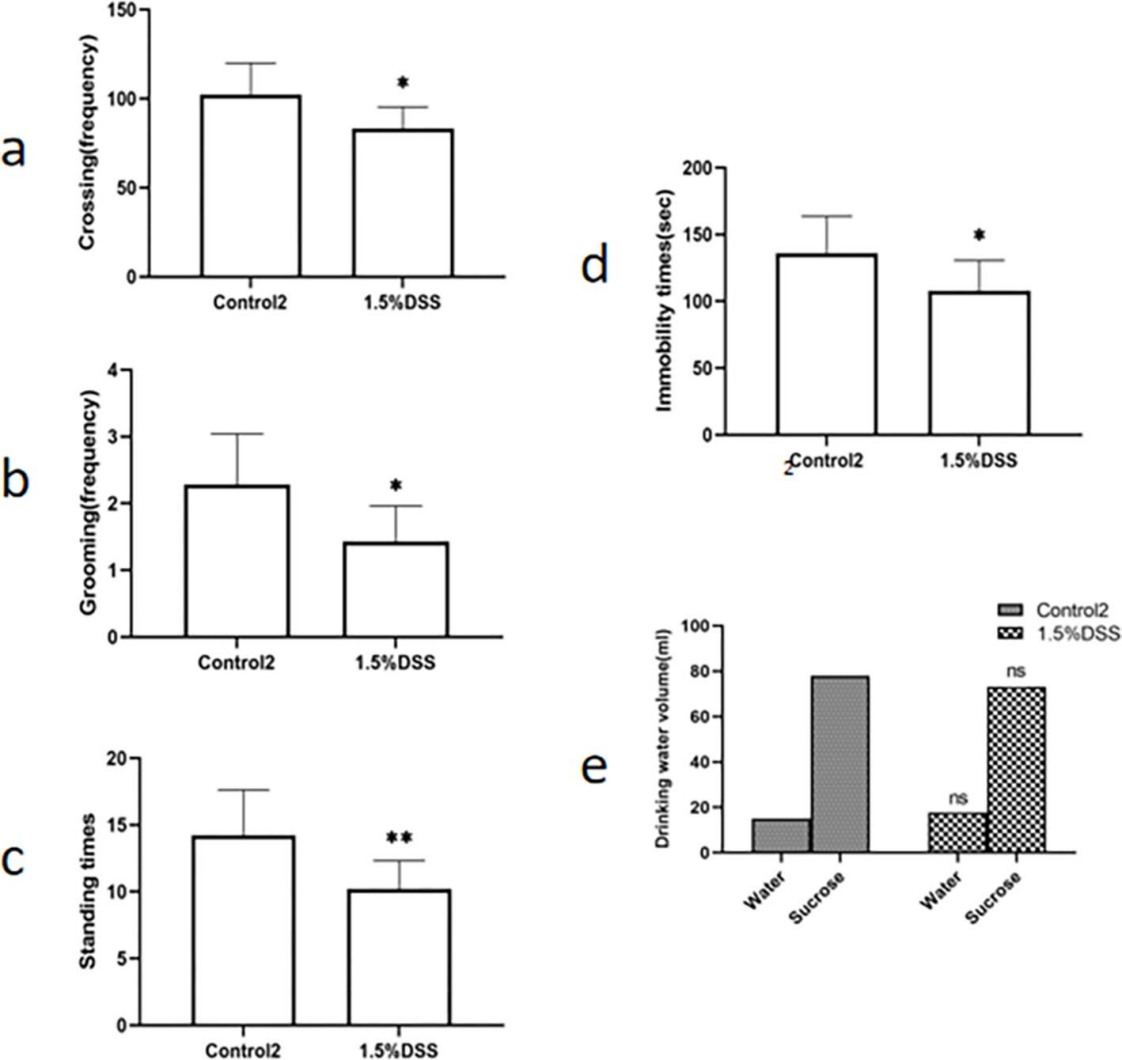
Behavior of mice in chronic UC model group. After the UC model was established, C57BL/6 mice were detected open field experiment, tail suspension experiment and sucrose preference experiment. **a** Running times, **b** grooming times, **c** standing times, **d** tail suspension resting time,  **e** sucrose preference. Data are expressed as mean ± SD of ten mice in each group. *P < 0.05 and **P < 0.01vs. Control2 group

1.5%DSS induced the decrease of activity and behavioral despair in mice (Fig. 4), which is manifested by the decrease of crawl number in the open field experiment, the prolongation of immobility time in the tail suspension experiment, sucrose preference experimental gap is not obvious. Different from the acute UC model induced by 3% DSS, the immobility time of chronic UC model mice induced by 1.5% DSS decreased significantly in the tail suspension test and forced swimming test. Therefore, the chronic UC model induced by 1.5% DSS can lead to significant behavioral changes in C57BL / 6 mice.

### Comparison of Hippocampal HE staining between acute UC model and chronic UC

**Fig. 5:**
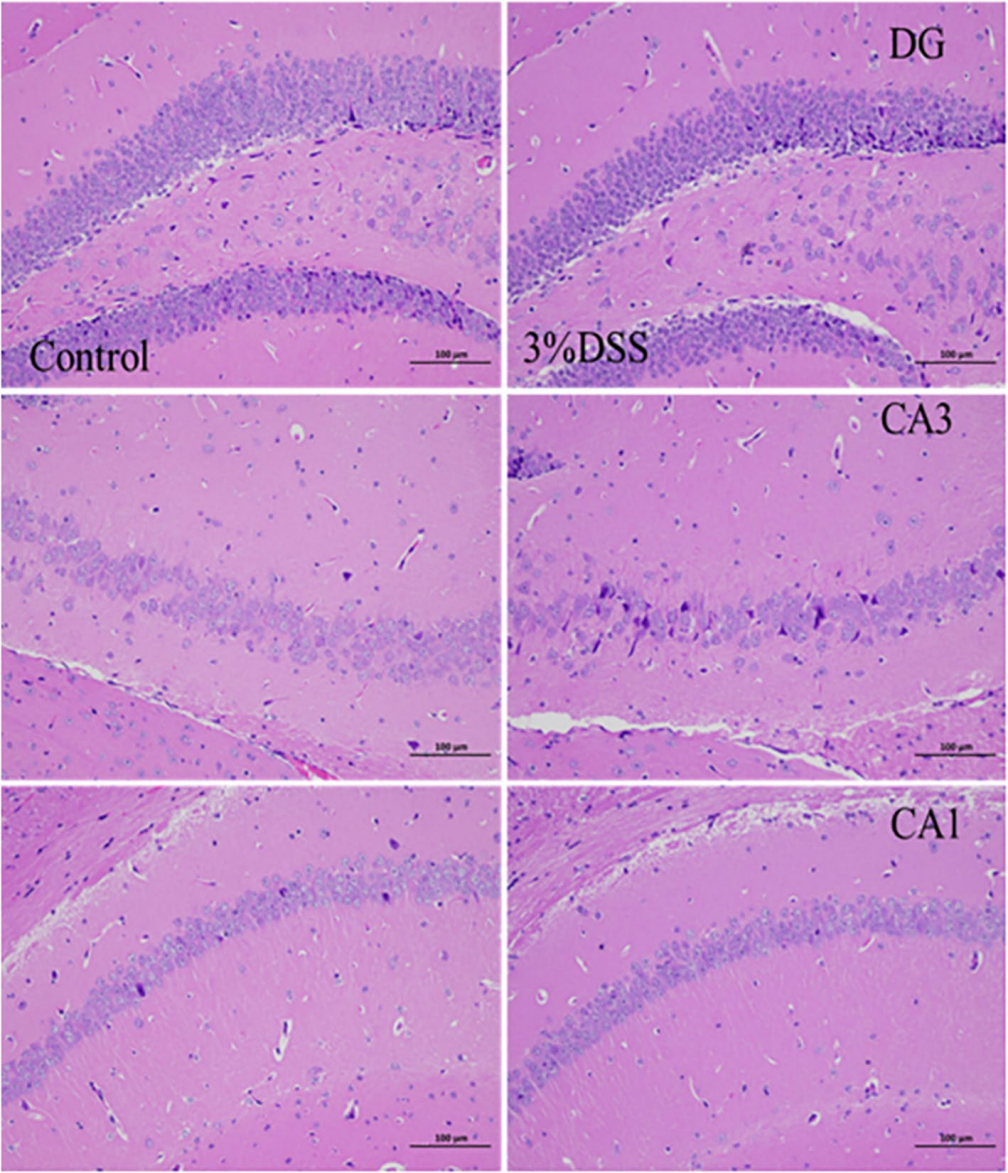
HE staining of hippocampus of mice in acute UC model group(200 ×)

**Fig. 6:**
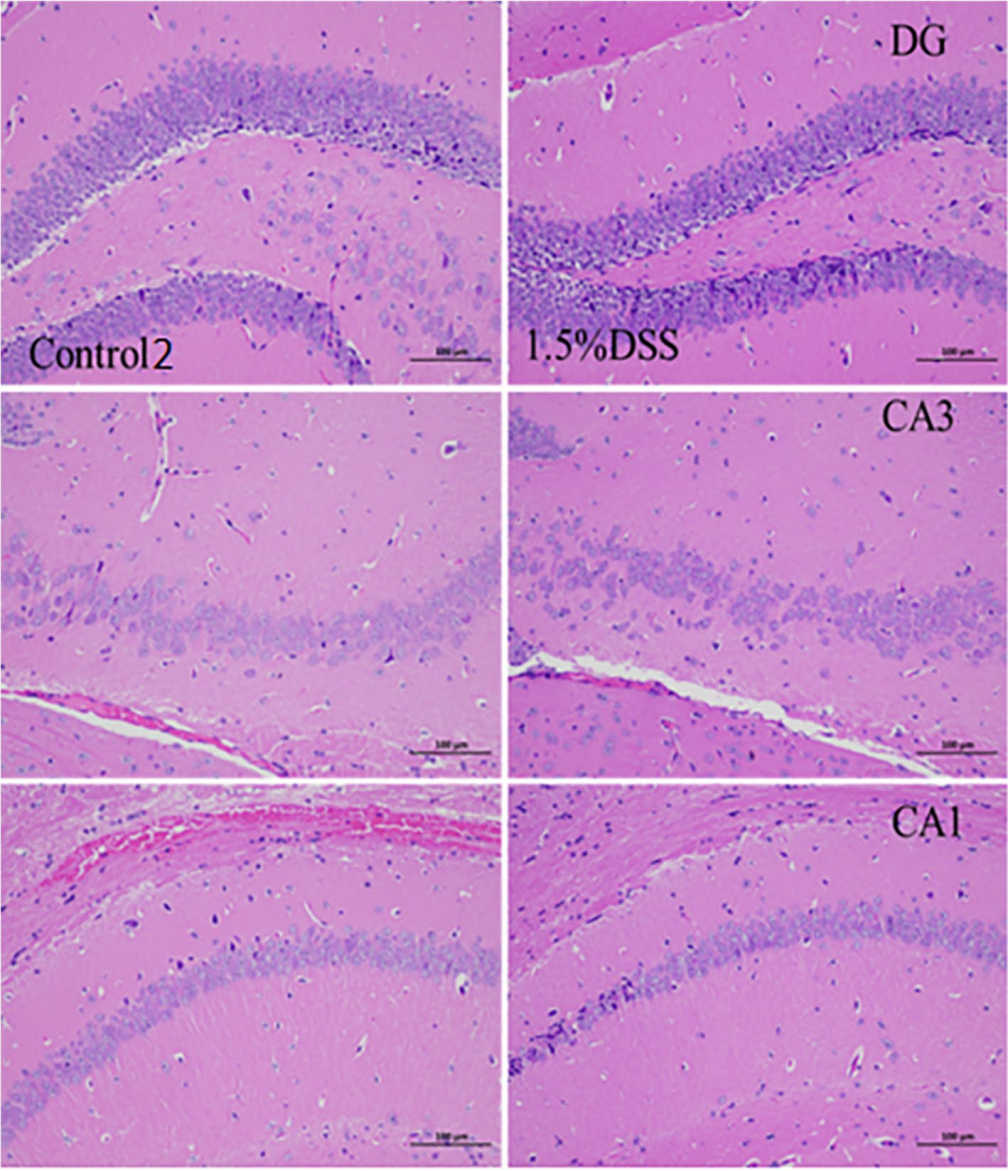
HE staining of hippocampus of mice in chronic UC model group(200 ×)

HE staining of hippocampus can observe whether the brain cells of mice are damaged. HE staining of the hippocampus of 3% DSS group mice showed that there was no obvious loosening and reduction of cells in the DG, CA1 and CA3 regions, and a small amount of nuclear pyknosis in the DG region (Fig. 5). However, the HE staining of the hippocampus of 1.5% DSS group mice showed that the cells in CA3 area were loosely arranged and the number of cells was reduced. There was a large amount of nuclear pyknosis in DG area and a small amount of nuclear pyknosis in CA1 area (Fig. 6). It shows that acute UC model has little effect on mouse brain cells, 9 while chronic UC model has certain effect on mouse brain cells.

## Discussion

UC is a chronic idiopathic inflammatory disease of the colon with unclear pathogenesis. Clinical epidemiology has found that most UC patients are chronic inflammatory bowel disease and are prone to mental abnormalities, usually manifested as depression, anxiety and other symptoms [22]. It shows that mental factors affect the pathogenesis of UC to a certain extent. According to the 2019 American College of Gastroenterology (ACG) guidelines for adult UC and the 2021 European Crohn’s and Colitis Organization (ECCO) guidelines, the commonly used drugs for the treatment of UC are 5-aminosalicylic acid and corticosteroids, immunosuppressive modulators, TNF drugs and monoclonal antibody drugs [26, 27]. These drugs mainly reduce the symptoms of UC patients by improving inflammation. It is difficult to completely cure UC, and these drugs have the disadvantages of inducing hepatitis, causing a serious infection, increasing the risk of lymphoma cancer, and being expensive [28, 29]. In laboratory UC drug research, the DSS-induced UC model has a short modeling time and a simple method and is widely used by researchers. Although clinicians have begun to use some antipsychotic drugs to adjuvant UC and achieved certain effects, few researchers have paid attention to its treatment mechanism [30–32]. Therefore, we guessed whether the mice in the DSS-induced UC model had behavioral abnormalities, and hoped to improve the behavioral abnormalities to achieve the purpose of treating UC.

We established acute and chronic UC models by DSS. The general condition, body weight, fecal state and other indicators of the mice were recorded during modeling, and whether the modeling was successful was judged by the changes in body weight, DAI score, colon length and colon HE staining results. The behavior of mice was detected by open field experiment, the tail suspension experiment, sucrose preference test and the levels of HE in the hippocampus were used to reflect whether the mice had mental disorders. The open-field experiment is usually used to measure the motor function and emotion of rodents. It has the characteristics of simple operation and good feasibility. It can evaluate the different behavioral changes of mice by recording the number of running times, the times of standing up, and the times of grooming. The number of runs and the number of uprights in the open field experiment reflected the rodent’s exploratory behavior in novel environments [33]. Studies have shown that fatigued or depressed rats have reduced voluntary movements, prefer to move around the edge of the box, and have less total travel distance. The tail suspension experiment is to induce depression and despair behavior by being unable to overcome the abnormal body position. It belongs to the "dry" behavioral despair model. Usually, immobility state latency and the immobility state duration percentage are used to evaluate the immobility state of the animal [34, 35].

By analyzing the body weight, DAI score, colon length and colon HE staining results of the mice, the mice in the 3% DSS model group and the 1.5% DSS model group had weight loss compared with the mice in the Control group and the Control2 group, blood in the stool, increased DAI score and shortened colon length. Colon HE staining showed that the colon tissue of the mice in the model group had crypt destruction, gland disorder, and inflammatory cell infiltration. The above results demonstrate that the modeling of acute UC model with 3% DSS and the chronic UC model with 1.5% DSS was successful. Through the analysis of the open field test, tail suspension test, forced swimming test, sugar water preference test and hippocampal HE staining results of mice, compared with the Control group, the mice in the 3% DSS model group had the number of running spaces, the number of time points in the open field test, and The number of hairs and the number of standing were reduced; in the tail suspension experiment, the resting time of the two groups of mice was not much different; in the sucrose preference test, the pure water drinking amount of the 3% DSS model group was slightly reduced while the sucrose water drinking The amount is greatly reduced. From the perspective of mouse behavioral indicators, the acute UC model induced by 3% DSS has a certain effect on the mental state of mice. However, through the analysis of the results of hippocampal HE staining, the hippocampal DG, CA1 and CA3 regions of the mice in the 3% DSS model group were not significantly different from those in the Control group, and the cells did not appear to be significantly loosening, reduced and pyknosis. Based on the analysis of various data and results, the acute UC model induced by 3% DSS has a certain effect on the behavior of mice, but it may be affected by UC symptoms rather than by mental factors. Compared with the Control2 group, the mice in the 1.5% DSS model group had reduced running times and standing times in the open field test, and the number of grooming was not much different; in the tail suspension experiment, the resting time of the mice in the 1.5% DSS model group was significantly reduced compared to the mice in the Control2 group; in the sugar water preference test, the difference between the two groups of mice was not significant. Therefore, the reason for the significant decrease of drinking sugar water in the 3% DSS group mice may be the pain and gastrointestinal discomfort caused by severe UC, which greatly reduced their drinking water. The UC symptoms of mice in the 1.5% DSS group were mild, so the drinking amount of purified water and sugar water had little change. The results of HE staining of hippocampus showed that a large number of pyknosis occurred in the DG area of the hippocampus of mice in the 1.5% DSS model group, a small amount of pyknosis occurred in the CA1 area, and the distribution of cells in the CA3 area was loose and significantly reduced. This indicated that the chronic UC model induced by 1.5% DSS had a certain effect on the nerve center of mice. Based on the analysis of various data results, the chronic UC model induced by 1.5% DSS may affect the behavior of mice through mental factors.

Therefore, we believe that the DSS-induced chronic UC model is an ideal model rather than an acute model when studying the relationship between psychiatric factors and the onset of UC. At the same time, the use of DSS-induced acute UC models to evaluate the efficacy of anti-UC drugs is insufficient, and more studies using chronic UC models should be considered. This is similar to the clinical course for UC patients.

## Conclusion

Our study shows that although the DSS-induced acute UC model can lead to behavioral abnormalities in C57BL/6 mice, it has no effect on mental aspects, and its behavioral abnormalities may be caused by the pathological characteristics of UC. DSS-induced chronic UC model makes C57BL/6 mice mentally abnormal, and the behavioral abnormality is caused by the influence of mental factors.

## Data Availability

All data generated or analysed during this study are included in this published article。
